# The mineralised epiglottis: a narrative review illustrated by an index case

**DOI:** 10.1007/s00276-026-03959-y

**Published:** 2026-08-11

**Authors:** Mugurel Constantin Rusu, Irina Elena Stăncioiu, Ilinca Maria Stăncioiu, Adelina Maria Jianu

**Affiliations:** 1 Division of Anatomy, Department 1, Faculty of Dentistry, “Carol Davila” University of Medicine and Pharmacy, 050474 Bucharest, Romania; 2 Department of Anatomy and Embryology, Faculty of Medicine, “Victor Babeş” University of Medicine and Pharmacy, 300041 Timişoara, Romania; 3 Municipal Emergency Clinical Hospital of Timişoara, 300041 Timişoara, Romania

**Keywords:** Epiglottis, Calcinosis, Larynx, Computed tomography angiography, Deglutition disorders

## Abstract

**Purpose:**

Epiglottic mineralisation is an uncommon anatomical variant whose embryological, histological, epidemiological, imaging and clinical implications are incompletely synthesised in the literature. We provide a narrative review of these topics, illustrated by an index case of almost complete epiglottic mineralisation identified on CT angiography.

**Methods:**

A case of incidentally detected epiglottic mineralisation on retrospective head-and-neck CT angiography was analysed using multiplanar reformations, maximum-intensity projections, bone-window images, and three-dimensional reconstructions. Measurements were obtained with calibrated workstation calipers and verified by two senior researchers in independent sessions.

**Results:**

In a 90-year-old man, almost complete mineralisation of the epiglottis involved the petiolus epiglottidis and extended through the infrahyoid portion (body and stalk) and the suprahyoid portion (body and base). The infrahyoid portion (body and stalk) measured 1.703 cm in height and the suprahyoid portion (body and base) 0.973 cm; the midline hyoid–epiglottis distance was 0.842 cm. The mineralised superolateral margins were 1.6 mm (right) and 1.4 mm (left) from the posterior pharyngeal wall; the unmineralised posterosuperior margin contacted it directly. No laryngeal-membrane mineralisation was identified.

**Conclusion:**

Almost complete epiglottic mineralisation is an uncommon anatomical variant of the laryngeal inlet. Extensive epiglottic involvement, angulation of the hyoid-epiglottic complex, near-contact and contact with the posterior pharyngeal wall, and absence of laryngeal membrane mineralisation make this case relevant to swallowing biomechanics, airway assessment, and the differential diagnosis of supraglottic calcific densities.

**Supplementary Information:**

The online version contains supplementary material available at 10.1007/s00276-026-03959-y.

## Introduction

The epiglottis is a leaf-shaped elastic-cartilage structure that guards the laryngeal inlet during deglutition. The thyroid, cricoid and most arytenoid cartilages are hyaline and show age- and sex-related ossification of the laryngeal skeleton [20, 41, 42]. By contrast, the elastic cartilage of the epiglottis has long been considered relatively resistant to mineralisation, a view reflected in older reports that described epiglottic calcification as rare [4, 27].

Cross-sectional imaging has refined this interpretation. Quantitative histomorphometry showed that calcium deposition in the adult human epiglottis increases with age, is greater in men and is most pronounced in the infrahyoid stalk region (petiolus epiglottidis) [21]. Postmortem and clinical CT studies now identify epiglottic calcification in a small but reproducible minority of adults, again with an older-male predominance [2, 25]. Extensive plate-like mineralisation involving the petiolus, the infrahyoid portion and the suprahyoid portion remains uncommon, and a dense supraglottic calcific structure may be misinterpreted as a foreign body or another calcified laryngeal element [6, 18].

Terminology is important. Calcification denotes deposition of calcium salts, whereas ossification implies organised bone formation with osteocytes, trabeculae or marrow. CT and CTA demonstrate mineral density but seldom prove bone histology; therefore, in the absence of histology, mineralisation is the most accurate term, and ossification should be reserved for histologically confirmed cases; imaging features such as corticated margins and trabecular architecture may be highly suggestive of ossification but remain presumptive in the absence of tissue sampling [27].

We present an index case of almost complete epiglottic mineralisation identified incidentally on CTA and use it as the basis for a narrative review of the embryology, histology, epidemiology, mechanisms, imaging features, differential diagnosis, and clinical implications of this entity.

## Index case

A retrospective head-and-neck CT angiography study from a 90-year-old man was reviewed after an epiglottic abnormality was noted incidentally on imaging. The CTA was performed for evaluation of cervico-cerebral arterial disease; no further clinical details were retrievable from the archived record. Swallowing status, aspiration or intubation history, and metabolic profile were unavailable, precluding assessment of whether systemic vascular or metabolic risk factors contributed to the mineralisation.

Image analysis was performed on a Horos DICOM workstation using multiplanar reformations, sagittal maximum-intensity projections, bone-window images, and three-dimensional reconstructions, such as in a previous anatomic variations report [35]. The CTA had been acquired on a 64-slice Siemens SOMATOM Definition AS scanner (Siemens Healthineers, Forchheim, Germany) at 120 kVp, with automatic tube-current modulation (CARE Dose4D; reference 150–200 mAs), a 64 × 0.6 mm detector configuration with z-flying focal spot, 0.5 s gantry rotation time, and pitch 0.9. Intravenous iohexol 350 mg I/mL was administered as a 70 mL bolus at 4 mL/s, followed by a 30 mL saline flush. Bolus tracking was performed in the ascending aorta using a 100 HU threshold. Axial datasets were reconstructed at 0.75 mm slice thickness and 0.5 mm increment with a medium-smooth vascular kernel (B26f).

**Fig. 1:**
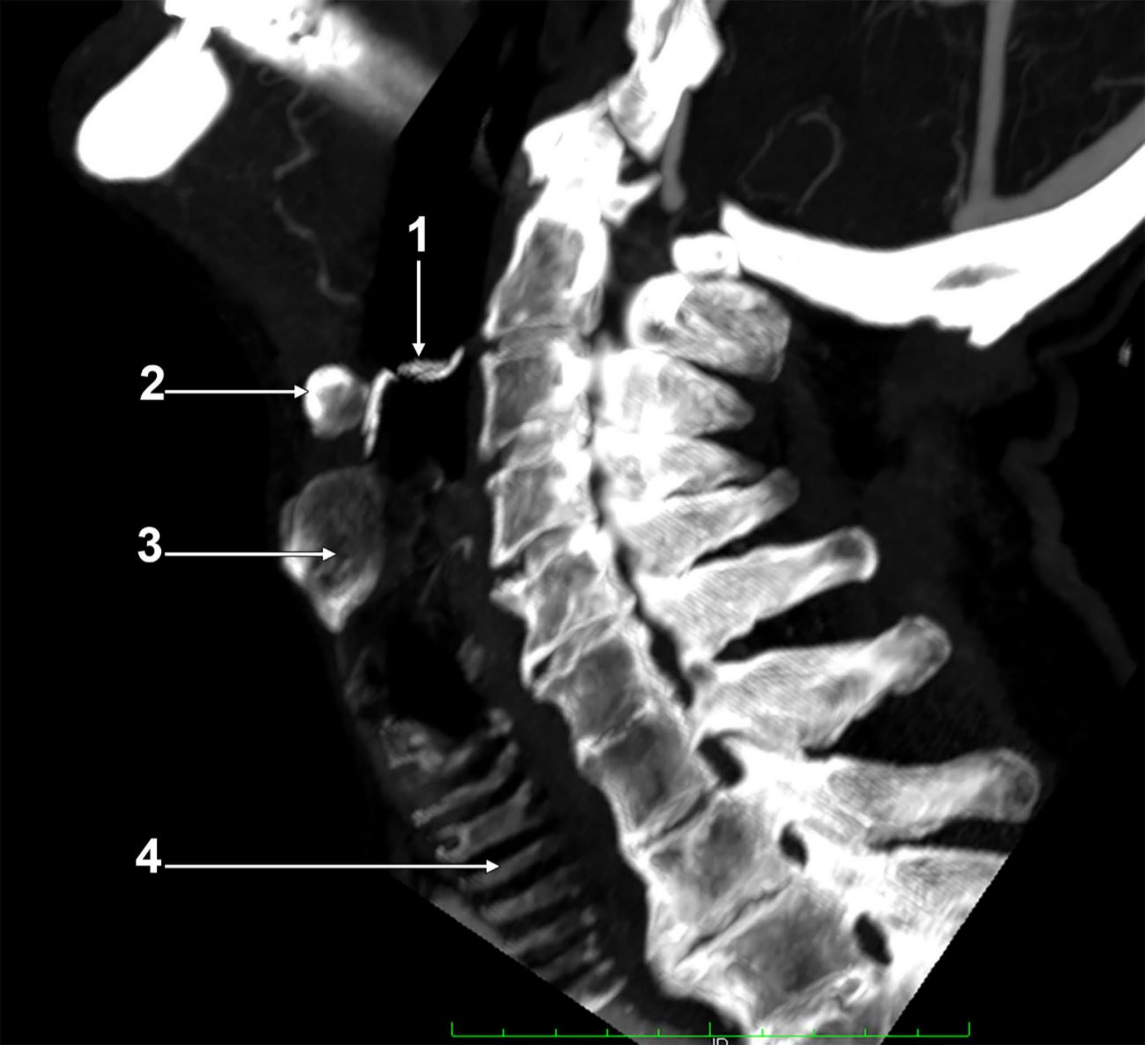
Sagittal CTA slice showing a mineralised epiglottis. (1) epiglottis; (2) hyoid body; (3) thyroid cartilage; (4) trachea

**Fig. 2:**
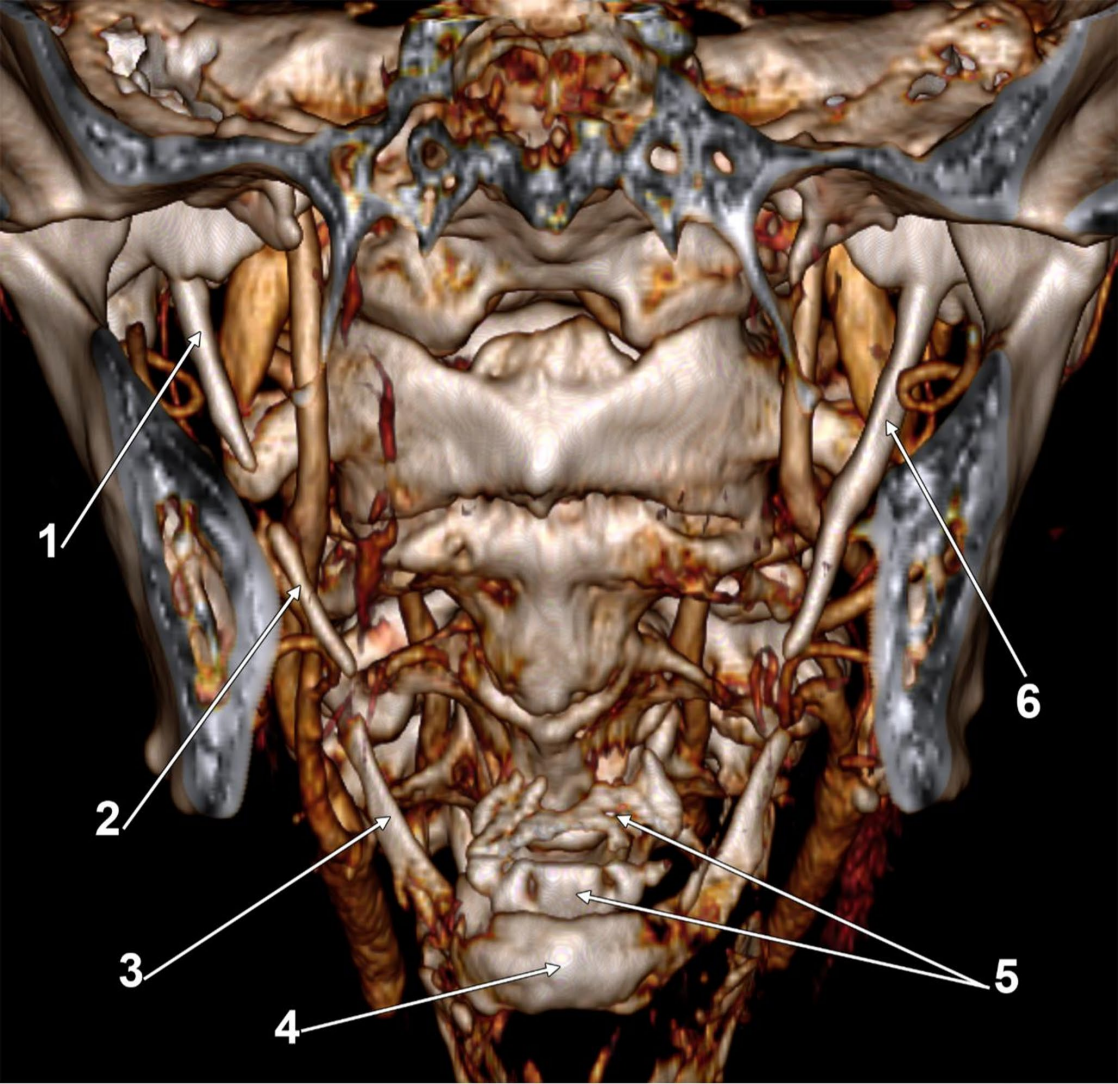
Three-dimensional volume rendering, anterior view. The body of the mandible was cropped out. (1) styloid process; (2) ossified keratohyal (stylohyoid ligament); (3) greater hyoid horn; (4) hyoid body; 5—mineralised epiglottis; 6. elongated styloid process

**Fig. 3:**
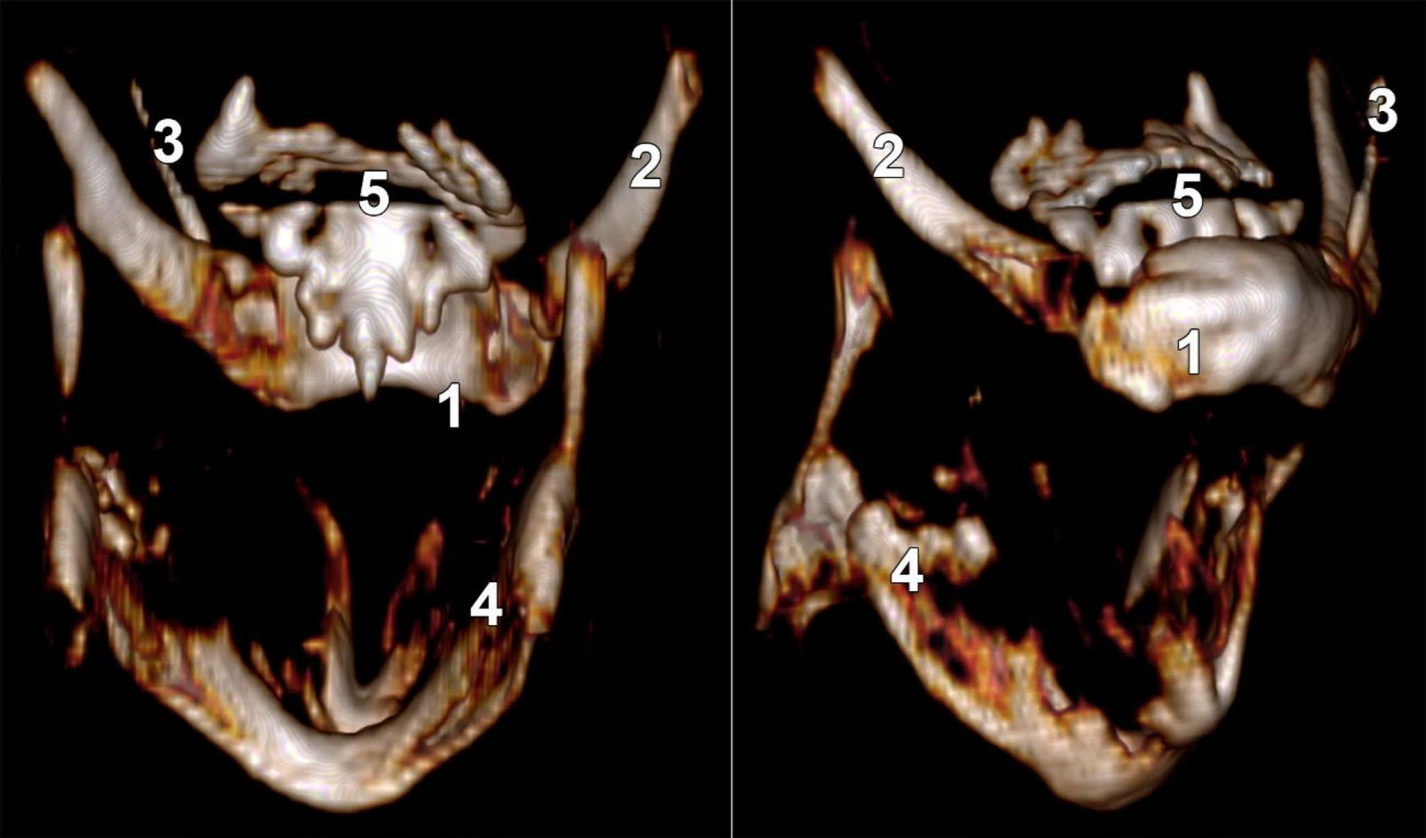
Three-dimensional rendering of the hyolaryngeal skeleton. (**A**) Posterior view. (**B**) Right anterolateral view. (1) hyoid body; (2) greater hyoid horn; (3) ossified hypohyal; (4) thyroid cartilage; (5) epiglottis

Sagittal, axial, and coronal reconstructions were examined to determine the anatomical origin of the mineralised structure and to exclude continuity with the hyoid bone, thyrohyoid ligament, or thyroid cartilage. Measurements were obtained directly from the DICOM images using the workstation calliper tool (Horos v3.3.6, open-source DICOM viewer) by a single observer. Each measurement was repeated in a separate session, and the mean value was used for reporting. All linear measurements are reported to one decimal place, consistent with the spatial resolution of CTA source images; sub-millimetre values should be interpreted with the understanding that the precision of caliper-based measurements on CT is constrained by voxel dimensions and partial-volume effects. Two senior researchers independently reviewed the CTA datasets. The final anatomical interpretation and measurements were established by consensus after joint verification of the multiplanar reconstructions. CTA demonstrated almost complete mineralisation of the epiglottis, involving the petiolus epiglottidis and extending through the infrahyoid portion (body and stalk) and the suprahyoid portion (body and base). The epiglottis was angled posteriorly at 90 degrees. This angle was measured on the midline sagittal reconstruction as the angle subtended between the long axis of the lower half (infrahyoid, stalk/petiolus) and the long axis of the upper half (suprahyoid, free margin) of the mineralised epiglottis, reflecting the degree of posterior curvature of the epiglottic plate at its mid-point. The hyoid body and mineralised epiglottis formed an angulated configuration with an anteroinferior vertical limb and a posterosuperior horizontal limb. (Fig. 1, Online Resource 1) (Fig. 2) (Fig. 3, Online Resource 2).

On the midline, the infrahyoid portion (epiglottic body and stalk, including the petiolus) measured 1.703 cm in height, and the suprahyoid portion (body and base) measured 0.973 cm. The midline hyoid–epiglottis distance was 0.842 cm. The mineralised superolateral margins approached the posterior pharyngeal wall, measuring 1.6 mm from it on the right and 1.4 mm on the left. At the same time, the unmineralised posterosuperior epiglottic margin contacted the posterior pharyngeal wall. The hyoid-greater-horn-to-thyroid-cartilage interval measured 1.00 cm on the right and 0.96 cm on the left. No mineralisation of the laryngeal membranes was identified, supporting an epiglottic-cartilage-centred process rather than diffuse laryngeal membranous or ligamentous mineralisation.

The measurements and their anatomical interpretation are summarised in Table 1.

Literature search. A narrative literature search was conducted in PubMed, Google Scholar, and Scopus using the terms “epiglottis calcification”, “epiglottis mineralisation”, “epiglottis ossification”, “laryngeal cartilage mineralisation”, and “epiglottis CT”, without date restriction, in English. Reference lists of retrieved articles were hand-searched for additional sources. No formal inclusion or exclusion criteria were applied; studies were selected for relevance to the topics covered in this review.

**Table 1:** CTA measurement summary and anatomical interpretation

Parameter	Measurement/finding	Anatomical interpretation
Demographics	Male, 90 years	Consistent with the older-male predominance reported in CT and histological series
Extent of mineralisation	Almost complete; petiolus, infrahyoid and suprahyoid portions	More extensive than small basal or punctate age-related calcification
Midline infrahyoid portion (body and stalk, incl. petiolus)	1.703 cm	Supports a plate-like epiglottic configuration
Midline suprahyoid portion (body and base)	0.973 cm	Confirms extension beyond the free margin or base
Midline hyoid–epiglottis distance	0.842 cm	Defines the relation between the mineralised epiglottis and the hyoid framework
Right superolateral margin to posterior pharyngeal wall	1.6 mm	Close approximation; relevant to swallowing mechanics and foreign-body mimicry
The left superolateral margin to the posterior pharyngeal wall	1.4 mm	Close approximation; near-symmetric
Posterosuperior (unmineralised) margin	Contacts the posterior pharyngeal wall	Direct contact requires clinical correlation
Hyoid-greater-horn-to-thyroid-cartilage interval	Right 1.00 cm; left 0.96 cm	Near-symmetric hyoid–laryngeal spacing
Laryngeal-membrane mineralisation	Absent	Favours an epiglottic-centred process rather than diffuse laryngeal mineralisation

## Review

### Embryology and developmental anatomy

The epiglottis develops within the pharyngeal-arch and hypobranchial-eminence complex and is integrated with the hyoid, tongue base and supraglottic larynx. Fetal histological studies identify the mesenchymal condensation of the epiglottic cartilage posterosuperior to the hyoid at approximately nine weeks, followed by descent of the inferior component towards the level of the thyroid cartilage [22]. After birth, the epiglottis and pre-epiglottic space change substantially from the neonatal period into adulthood as the vocal tract and swallowing apparatus mature [36]. This developmental integration is relevant because the epiglottis is a mobile, elastic element connected to the hyoid apparatus, the tongue base, the valleculae, the aryepiglottic folds, the laryngeal inlet, and the pre-epiglottic space. Mineralisation may therefore have functional relevance even when detected incidentally.

Histological studies of the epiglottis and their supporting ligaments in older adults have clarified the structural basis for age-related functional decline. Serikawa et al. (2023) examined 20 cadaveric specimens (ages 64–94, mean 79.8 years) using haematoxylin and eosin, Elastica Van Gieson, and immunohistochemical staining (anti-fibrillin-1 and anti-von Willebrand factor). They observed glandular atrophy with fatty replacement, connective tissue fibrosis characterised by increased collagen and reduced elastic fibres, and fibrillin-1-positive oxytalan microfibrils around vascular walls within the epiglottic ligaments. Collagen fibres ran toward and through the cartilage, whereas the mesh-like elastic fibres discontinued in front of the cartilage. In specimens from tube-fed subjects – presumed to have had no oral intake – oxytalan fibre content was lower than in orally-fed subjects, suggesting that disuse reduces the elastic microfibril network that supports epiglottic stretchability during deglutition. The authors concluded that the decrease in elastic fibres and the increase in collagen fibres represent age-related changes that may impair epiglottic descent and contribute to aspiration in older adults [37]. These findings provide a histological context for the resistance of the elastic epiglottis to the mineralisation pathways that affect hyaline cartilage: the elastic fibre network, when intact, may mechanically and biochemically inhibit the calcium deposition that characterises dystrophic mineralisation.

### Histological composition and mineralisation terminology

The predominantly elastic composition of the epiglottis distinguishes it from the predominantly hyaline thyroid, cricoid and arytenoid cartilages (noting that the apical and vocal-process portions of the arytenoid cartilages are elastic and share the relative resistance to mineralisation characteristic of elastic tissue [26]), which explains why epiglottic mineralisation has historically been considered unusual. Hyaline laryngeal cartilages mineralise in a recognised sequence that becomes common in older adults [28, 41], whereas the elastic epiglottis has been treated as an exception. Quantitative analysis nevertheless shows that calcium deposition in adult epiglottic cartilage increases with age, is more pronounced in men, and predominates in the infrahyoid stalk region (petiolus epiglottidis) [21]. The distinction between calcification and ossification remains important: imaging-based laryngeal cartilage mineralisation has been graded as calcification, sclerosis or ossification [43], but CT alone cannot establish bone histology. Mineralisation is therefore the appropriate descriptor in the present case, and ossification should be reserved for histological confirmation or imaging with clear trabecular architecture [27]. Malkidou and Fiska (2026) reported CT attenuation comparable to cortical bone in four of five cases, suggesting advanced mineralisation, while acknowledging that histological distinction requires tissue sampling [25].

The claim that the epiglottis does not mineralise has a long textual pedigree that merits critical scrutiny. The most direct and influential statement comes from Fernandez-Rodriguez et al. (2009), who repeated the classical position verbatim: “En los textos clásicos se afirma que el cartílago epiglotico es el único que, por sus propiedades elásticas, no sufre esta transformación ósea” (“In the classical texts it is stated that the epiglottic cartilage is the only one that, by virtue of its elastic properties, does not undergo this bony transformation”) [11]. Stojanovic (2013), in a chapter on laryngeal anatomy, stated categorically that “the epiglottic cartilage is elastic and never ossifies in contrast to others that are hyaline and therefore start to ossify right after puberty” [39]. Similarly, Joshi et al. (2012), in a widely-cited imaging review, wrote that “the epiglottis and the vocal process of arytenoids are fibrocartilages that do not ossify”, explicitly using this as the basis for the expected CT appearance of the epiglottis as soft-tissue attenuation without mineralisation [19]. Hately et al. (1965), whose 516-case radiographic series remains one of the most comprehensive studies of laryngeal ossification, did not examine the epiglottis as a separate structure and implicitly excluded it from the ossification sequence by restricting their analysis to the thyroid, cricoid, and arytenoid cartilages; the epiglottis does not appear in their ossification patterns or figures [15]. Taken together, these sources reflect a consistent pre-CT consensus rooted in the elastic nature of the epiglottic cartilage. Elastic cartilage contains a type-II collagen and proteoglycan matrix similar to hyaline cartilage, but is distinguished by an abundant network of elastic fibres and oxytalan microfibrils within the pericellular and interterritorial matrix. This elastic fibre architecture — rather than an absence of collagen-II — was thought to confer relative resistance to mineralisation, by providing mechanical resilience against the deformation-related calcium nucleation that drives age-related mineralisation in hyaline cartilage, and by maintaining a perichondrial and matrix microenvironment less permissive to hydroxyapatite deposition. The consensus was not, however, based on systematic large-scale CT data, and the cases reported by Ardran (1965), Milroy (1992), Kano et al. (2005), Ampanozi et al. (2021) and Malkidou and Fiska (2026) have progressively eroded it [2, 4, 21, 25, 27]. Notably, Girit and Senol (2019) described CT-proven calcification of all laryngeal cartilages, including the epiglottis, in a 14-year-old patient with Keutel syndrome, demonstrating that even in childhood, and even in the epiglottis, mineralisation can occur when the molecular inhibition of ectopic calcification (mediated by matrix Gla protein, MGP) is disrupted [13]. Ebrahim and Shaik (2023) reported CBCT-detected epiglottic calcification in a 70-year-old man and explicitly noted that “calcification of the epiglottis has been rarely documented and is poorly understood”, confirming that the entity remains under-recognised in clinical imaging practice [9]. The accumulated evidence therefore warrants replacing the categorical statement that the epiglottis does not mineralise with the more accurate position that epiglottic mineralisation is uncommon but definite, age- and sex-related, and mechanistically distinct from the ossification of hyaline laryngeal cartilages.

### Epidemiology and prevalence

Older literature regarded the elastic epiglottis as essentially non-calcifying. In a radiographic survey of several thousand examinations, Baclesse (1938, cited in Ardran, 1965) identified only five cases of fragmentary epiglottic calcification, and Ardran (1965) found no epiglottic calcification across several thousand larynges examined in life and at postmortem, apart from one well-calcified case [4]. Modern CT series show a low but definite prevalence: a postmortem CT series of 2,930 cases reported calcified epiglottis in 4.1%, associated with older age and male sex [2], and a retrospective neck-CT study of 203 living adults reported epiglottic cartilage calcification in 2.46%, exclusively in older males with a median age of 75 years [25]. The difference relative to historical studies probably reflects the greater sensitivity of cross-sectional imaging.

**Fig. 4:**
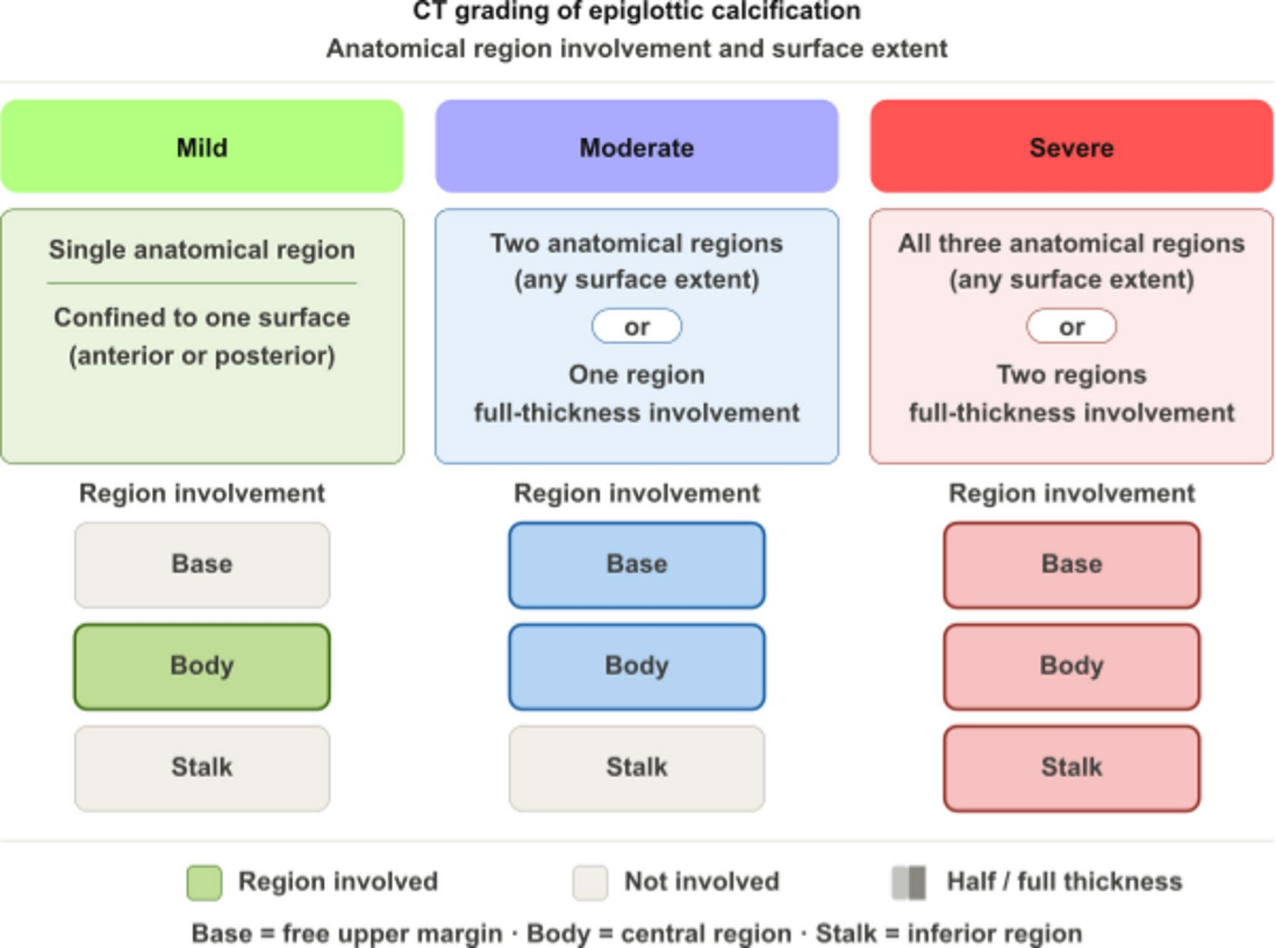
Semi-quantitative CT grading system for epiglottic cartilage calcification. The epiglottis is divided into three anatomical regions: the Base (free upper margin, posterior to the tongue base), the Body (central region at the level of the hyoepiglottic ligament insertion), and the Stalk (inferior region at the thyroepiglottic ligament insertion). Calcification is graded by the number of regions involved and the surface extent of involvement within each region. Mild: calcification limited to a single region, confined to one surface (anterior or posterior). Moderate: calcification involving two anatomical regions (any surface extent), or one region with full-thickness (transmural) involvement. Severe: calcification affecting all three anatomical regions (any surface extent), or two regions with full-thickness involvement. Coloured zones indicate involved regions (green: mild; blue: moderate; dark red: severe); grey zones indicate no involvement. The present case, with almost complete mineralisation of the petiolus with both infrahyoid and suprahyoid portions involved, would exceed the severe grade as defined for this system. After Malkidou N, Fiska A. Anat Cell Biol. 2026;59:54–58

Malkidou and Fiska (2026) proposed a semi-quantitative CT grading system: mild calcification limited to a single region (base, body or stalk) and one surface; moderate calcification involving two regions or one region with full-thickness involvement; and severe calcification involving all three regions or two regions with full-thickness involvement [25] (Fig. 4). In their living cohort, all five cases were mild or moderate, without stalk involvement or associated epiglottic deformity. In published cases summarised by the same authors, severe grades involving the full thickness of the body and base were associated with metabolic disease, particularly end-stage renal disease with secondary hyperparathyroidism or renal transplantation [18, 34].

Epiglottic calcification remains much less frequent than mineralisation of the hyaline laryngeal skeleton, which increases with age, shows a male predominance and is often asymmetric between cartilages and sides [3, 28, 40, 41, 43]. Reported prevalence and age/sex patterns are summarised in Table 2. Small basal epiglottic calcifications are therefore not necessarily reportable in isolation; the present case is notable for its plate-like, almost complete extent, angulated configuration and relation to the posterior pharyngeal wall.

**Table 2:** Reported prevalence of epiglottic and laryngeal cartilage mineralisation. Historical radiographic surveys and modern CT series are not directly comparable owing to differences in method and sensitivity; figures are reported as in the source studies. † Cited in Ardran (1965); original not independently retrieved

Study (year)	Method/population	*n*	Reported finding	Sex/age
Baclesse (1938)†	Radiographic survey	several thousand	Fragmentary epiglottic calcification in only 5 cases (< 0.1%)	–
Ardran (1965) [4]	Radiography in life and at postmortem	“some thousands”	None detected across the series; single calcified case reported (68-year-old man)	male (index case)
Kano et al. (2005) [21]	Quantitative histomorphometry (autopsy)	–	No prevalence figure; calcium deposition rises with age, greatest in the infrahyoid stalk region (petiolus epiglottidis)	↑ age; male
Ampanozi et al. (2021) [2]	Postmortem CT	2,930	Calcified epiglottis in 4.1%	older age; male
Malkidou and Fiska (2026) [25]	Retrospective adult neck CT (living cohort)	203	Epiglottic calcification in 2.46% (5/203); mild–moderate grade; no stalk involvement; no associated deformity	older males; median age 75
*Broader laryngeal skeleton (hyaline cartilages; context only*,* not epiglottis-specific)*				
Turk and Hogg (1993) [41]	Cadaver/autopsy radiology + histology, ages 14–101	48	Calcification in all but the 14- and 20-year-old specimens (≈ universal in adults)	minor sex differences
Sycińska-Dziarnowska et al. (2024) [40]	Lateral cephalograms	957	Reaches 100% by age 30 (women) / 50 (men); detectable earlier	earlier/higher in women
Miyamoto et al. (2022) [28]	Ultra-high-resolution CT	47	Thyroid ossification onset < 20 years; degree increases with age	male > female
Mupparapu and Vuppalapati (2005) [29]	Lateral cephalograms, ages 10–59	359	Thyroid and cricoid ossification frequencies tabulated (thyroid > cricoid)	male predominance
Zan et al. (2011) [43]	Neck CT, patients without laryngeal cancer	–	Asymmetric arytenoid mineralisation is common in cancer-free patients.	–

### Mechanisms of mineralisation

In an older patient, the most plausible mechanism is age-related dystrophic calcification of elastic cartilage, with loss of elastic fibres, chondrocyte degeneration, fibrosis and local calcium deposition [21]. General cartilage mineralisation pathways include matrix vesicle formation, alkaline phosphatase activity, and hydroxyapatite nucleation. However, these mechanisms have been studied primarily in the growth plate and hyaline cartilage rather than in elastic epiglottic cartilage [1, 5, 37]. In laryngeal cartilages, matrix-vesicle-mediated mineralisation can occur together with a collagen-fibril-guided pattern of crystal growth [8]; however, those observations derive from hyaline thyroid cartilage, and their applicability to the elastic epiglottis remains uncertain.

Histological evidence from Serikawa et al. (2023) adds a complementary tissue-level perspective. Their observation that collagen fibre content increases and elastic fibre content decreases in the epiglottis with ageing is consistent with the progression from an elastic to a more fibrotic matrix. This microenvironment may facilitate calcium nucleation and hydroxyapatite deposition by reducing the mechanical impedance that elastic fibres normally provide against mineralisation. The reduction in oxytalan fibres (fibrillin-1-positive microfibrils) in tube-fed subjects further suggests that disuse accelerates the loss of the elastic scaffold, potentially lowering the threshold for dystrophic calcification. This mechanism is distinct from the endochondral ossification of hyaline cartilage. It may explain why epiglottic mineralisation, when it occurs, tends to be patchy and plate-like rather than following a trabecular pattern [37].

Systemic mineral metabolism should also be considered. In the cases reported by Gunbey et al. (2014) and Seth et al. (2024), both presenting with clinically significant dysphagia in men aged 57 and 91 years, serum calcium, phosphorus and parathyroid hormone were within normal limits, supporting a dystrophic mechanism without systemic metabolic perturbation [14, 38]. In contrast, Jeph et al. (2017) reported diffuse epiglottic calcification in a 55-year-old man with end-stage renal disease and secondary hyperparathyroidism, in whom the parathyroid hormone level was 1,472 pg/mL (reference range 10–65 pg/mL); a neck CT nine years earlier had been normal [18]. Epiglottic calcification has also been described with soft-tissue amyloidosis [33], renal transplantation [34] and granulomatous disease [13, 32]. Local pharyngeal hypomotility, for example, from a coexisting Zenker diverticulum, has been proposed as a biomechanical predisposing factor by reducing the repetitive mechanical stimulus to epiglottic movement during swallowing. Extensive mineralisation, younger age, or atypical morphology should therefore prompt review of calcium, phosphate, parathyroid hormone, vitamin D, and renal function, and consideration of prior radiotherapy, local trauma, inflammation, or granulomatous disease.

### Imaging features, grading, and reporting

CT and CTA are the most useful modalities for detecting and characterising epiglottic mineralisation. Reports should specify location (free margin, body, base or petiole), morphology (punctate, nodular, laminar, plate-like or bulky), extent, and whether the finding is isolated or associated with broader laryngeal-cartilage ossification, which CT can depict and grade [42]. Bone-window source images are preferable to thick MIP images when excluding superimposition. The semi-quantitative CT grading scheme proposed by Malkidou and Fiska (2026) provides a practical framework for structured reporting [25]. (Fig. 4)

### Differential diagnosis

The main mimics are a calcified foreign body, hyoid-horn projection, ossified thyrohyoid ligament, superior horn of the thyroid cartilage, triticeous cartilage calcification, arytenoid, corniculate or cuneiform mineralisation, post-radiotherapy chondronecrosis, granulomatous calcification, chondroma, and low-grade chondrosarcoma. On CT, epiglottic calcification typically appears as submucosal calcification without a mucosal break or inflammatory change. In contrast, a foreign body is usually focal, projects into the oropharyngeal lumen and may be associated with adjacent mucosal disruption [18]. Foreign-body mimicry is clinically important because affected patients may present with acute dysphagia or globus sensation [6, 18]. Asymmetric or irregular mineralisation of the laryngeal cartilages also occurs in individuals without malignancy and should not be overcalled as tumour invasion or pathological sclerosis [43]. Reports of incidental and symptomatic epiglottic calcification reinforce the value of recognising this entity on imaging [34].

### Swallowing biomechanics and clinical presentation

Epiglottic inversion during swallowing depends on coordinated hyolaryngeal excursion, tongue-base retraction, pharyngeal pressure, laryngeal elevation and the elasticity of the epiglottis itself. A heavily mineralised, less compliant epiglottis may result in reduced retroflexion, and symptomatic cases with foreign-body sensation, dysphagia, abnormal epiglottic mobility, and aspiration have been reported [14, 18, 38]. Flexible nasolaryngoscopy in symptomatic patients has shown a posteroinferiorly translocated, thickened epiglottis with reduced mobility during swallowing, stasis in the pyriform sinuses and displacement of the free epiglottic edge towards the posterior pharyngeal wall [14, 38]. These findings parallel the posterior pharyngeal wall contact documented on CTA in the present case. A barium swallow examination may be normal despite CT-detected calcifications and endoscopic restriction of mobility [38], supporting the use of cross-sectional imaging and endoscopy when symptoms persist. Clinical presentation ranges from liquid aspiration to globus sensation without dysphagia, as illustrated by Jeph et al. (2017), in which the patient suspected an impacted fish bone [18]. Epiglottic calcification has therefore been considered a diagnosis of exclusion in elderly patients with oropharyngeal dysphagia when standard clinical and radiological evaluation identifies no other cause [38].

Causality should not be assumed. Dysphagia in older patients is multifactorial, and abnormal epiglottic movement can also reflect impaired hyoid mobility, tongue-base dysfunction, neurological disease or cervical osteophytes. In symptomatic patients, correlation with flexible laryngoscopy, fibreoptic endoscopic evaluation of swallowing (FEES) or videofluoroscopy is appropriate, and mechanical analyses confirm that impaired epiglottic inversion is multifactorial [31].

A mechanistically analogous situation is described by Lewis et al. (2015) in a case of Eagle’s syndrome (ES) presenting with moderate-to-severe pharyngeal dysphagia. Their 59-year-old male patient had CT-confirmed ossification and ankylosis of the stylohyoid ligament with pseudoarticulation of the hyoid to the thyroid cartilage, causing tethering of the hyolaryngeal complex. This morphological constraint – reduced laryngeal excursion due to structural rigidity rather than intrinsic epiglottic stiffness – produced a pattern of pharyngeal dysphagia with supraglottic penetration and pyriform sinus stasis closely resembling the dysphagia reported in symptomatic epiglottic mineralisation. Pre- and postoperative fibreoptic endoscopic evaluation of swallowing (FEES) with Penetration Aspiration Scale scoring documented significant improvement in laryngeal elevation and pharyngeal constriction following surgical release of the ossified ligament. However, recovery was delayed and required structured pharyngeal strengthening exercises targeting laryngeal elevation [24]. The parallel with epiglottic mineralisation is instructive: in both conditions, the structural rigidity of a component of the hyolaryngeal complex impairs epiglottic inversion, and in both, rehabilitation requires not just the removal of the mechanical obstruction but active neuromuscular retraining of the swallowing musculature.

### Airway considerations

Airway implications should be interpreted in a case-specific manner. In the largest CT prevalence study, epiglottic calcification was not significantly associated with failed endotracheal intubation [2], and extensive epiglottic mineralisation is not an established independent predictor of intubation failure. The present case nevertheless has features that may matter during airway instrumentation: plate-like rigidity of the epiglottis, an angulated hyoid-epiglottic configuration and superolateral margins located within 1.4–1.6 mm of the posterior pharyngeal wall.

During direct laryngoscopy, the epiglottis is displaced anteriorly, either indirectly through the vallecula with a Macintosh blade or by direct contact with a Miller blade. A mineralised, plate-like epiglottis may resist displacement, transmit force atypically or be more vulnerable to direct contact injury. Epiglottic haematoma is a rare but recognised complication of tracheal intubation, including in cases of technically straightforward intubation with a Cormack-Lehane grade I view. It may present several days after intubation with sore throat, hoarseness and dysphagia, and may progress to airway obstruction requiring ICU-level management or emergency tracheotomy [16]. Anticoagulant use, common in elderly patients, is an additional risk factor for epiglottic haematoma independent of intubation difficulty [16]. Abnormal laryngeal anatomy is a recognised risk factor for post-intubation laryngeal injury, and direct mechanical trauma to the epiglottis is an important mechanism for supraglottic lesions after instrumentation [17].

Technique selection may also matter. In bougie-assisted intubation, the gum-elastic bougie is directed anteriorly under the epiglottis and into the trachea before the endotracheal tube is advanced over it [10]. In the present case, the narrow residual space between the mineralised epiglottic cornua and posterior pharyngeal wall (1.4–1.6 mm bilaterally) could limit the working space for this manoeuvre. Video laryngoscopy, preferred for anticipated difficult airways because it improves first-attempt success across operator experience levels [10], may improve epiglottic visualisation and reduce the need for direct epiglottic contact compared with Miller-blade direct laryngoscopy. When extensive epiglottic mineralisation is identified before induction, communication with the anaesthetic team and advance preparation of a defined back-up strategy are prudent.

### Epiglottic mineralisation and obstructive sleep apnea: a proposed mechanism

The relationship between epiglottic mineralisation and obstructive sleep apnea (OSA) has not previously been discussed in the literature. Yet, the present case provides a compelling mechanistic basis for such a link. The epiglottis is an established, though historically underappreciated, site of upper airway collapse in OSA. Drug-induced sleep endoscopy (DISE) studies have found epiglottic collapse in up to 16.4% of OSA patients, with four recognised patterns: anterior-posterior collapse with a rigid (“trapdoor”) epiglottis (48%); anterior-posterior collapse with a lax (“floppy”) epiglottis (13.5%); lateral-lateral collapse with an omega-shaped (“book”) epiglottis (14.5%); and secondary collapse driven by adjacent structures (24%) [7, 12]. A fully mineralised epiglottis represents the extreme of the “rigid trapdoor” spectrum: it is structurally a bony plate that cannot be deflected, recoiled, or vary its position dynamically. The clinical implication is that, unlike a floppy epiglottis in which CPAP-generated positive pressure may paradoxically worsen obstruction by pushing the structure further into the laryngeal inlet [12], a heavily mineralised epiglottis may represent a predominantly fixed rather than dynamic obstruction – one that may be unlikely to respond to CPAP if the structure is already approximating the posterior pharyngeal wall, though this hypothesis requires prospective clinical testing.

The 90° posterior angulation in the present case amplifies this risk through three independent mechanisms. First, at 90°, the epiglottis is essentially horizontal at rest – already oriented perpendicular to the airway axis rather than the normal oblique position of approximately 40–50°. The CTA measurements demonstrating the superolateral margins at only 1.4–1.6 mm from the posterior pharyngeal wall in the upright waking position confirm that obstruction may already exist; in the supine sleeping position, posterior gravitational displacement of the tongue base and pharyngeal soft tissues would further reduce or eliminate this residual gap. Second, hyoid position is a central determinant of OSA severity: an inferiorly and posteriorly displaced hyoid is the most consistent cephalometric feature of OSA, and dynamic posterior-inferior hyoid movement during sleep is an independent predictor of epiglottic collapse, with epiglottic length above 16.6 mm being the most sensitive anatomical cut-off for collapse risk [23, 30]. In our case, the angulated hyoid-epiglottis configuration means that any posterior hyoid displacement during sleep – a normal OSA-related phenomenon – would geometrically rotate the mineralised horizontal limb further into the posterior pharyngeal space rather than away from it, the mechanical opposite of what occurs with a normally angled epiglottis. Third, the structure is irreversibly rigid: unlike dynamic epiglottic collapse, which is cyclical and can partially recover during expiration, the mineralised epiglottis at 90° would produce sustained rather than intermittent pharyngeal narrowing, potentially generating prolonged obstructive events with atypical polysomnographic signatures. Whether this patient had OSA is unknown – polysomnography was not performed – and at 90 years of age, multiple concurrent contributors to sleep-disordered breathing would be expected. Nevertheless, the morphological features in this case constitute a specific, previously undescribed anatomical substrate for OSA. We hypothesise that patients with extensive epiglottic mineralisation, particularly those with posterior angulation approaching or exceeding 90°, may warrant sleep-study evaluation if symptoms of sleep-disordered breathing are present, and that DISE in such patients may reveal a predominantly rigid obstruction pattern that differs from dynamic epiglottic collapse and may respond poorly to CPAP alone — considerations that require prospective clinical investigation.

### Management

Most cases are incidental and require no treatment. Symptomatic patients may benefit from ENT examination, flexible nasolaryngoscopy, swallow assessment and speech-language pathology input. In two published symptomatic cases with normal metabolic profiles, conservative measures were useful: chin-tuck head flexion during swallowing, small bolus volumes and short, frequent meals [14, 38]. These postures may reduce posterior displacement of the epiglottis towards the pharyngeal wall and improve bolus redirection through the hypopharynx. Aetiological work-up is appropriate in younger patients, in extensive mineralisation or when renal or endocrine disease is present. Surgery should be reserved for exceptional refractory cases with objective epiglottic immobility and aspiration; proposed options include supraglottoplasty or epiglottopexy [14, 38].

The experience of Lewis et al. (2015) with Eagle’s syndrome rehabilitation reinforces several principles applicable to the management of symptomatic epiglottic mineralisation. First, resolution of the mechanical obstruction – whether by surgical release of ossified stylohyoid ligament or, hypothetically, by supraglottoplasty in epiglottic mineralisation – does not immediately restore swallow function; pharyngeal dysphagia may persist postoperatively due to disuse-related muscular weakness. Second, targeted pharyngeal strengthening exercises focusing on laryngeal elevation and pharyngeal constriction, delivered by a speech-language pathologist, produced progressive improvement over six months and ultimately allowed resumption of a full oral diet. Third, the use of validated objective measures (FEES with Penetration Aspiration Scale scoring) at regular intervals guided diet consistency upgrades and confirmed genuine functional improvement. These principles suggest that symptomatic epiglottic mineralisation managed surgically should incorporate structured postoperative swallowing rehabilitation rather than relying on spontaneous recovery [24].

### Limitations

Histological confirmation was not available, so the finding is reported as mineralisation rather than ossification. Clinical, swallowing and metabolic data were unavailable, limiting functional correlation; the relation between posterior-pharyngeal-wall contact and symptoms cannot be established from the present data. Multiplanar source images should be included in the final report to confirm the epiglottic origin of the mineralised structure and to exclude the mimics discussed above.

## Conclusion

This narrative review, illustrated by an index case of almost complete epiglottic mineralisation in a 90-year-old man, synthesises the embryology, histology, epidemiology, mechanisms, imaging, differential diagnosis, swallowing biomechanics, airway implications, and clinical management of this underrecognised entity. The long-standing textbook assertion that the epiglottis does not mineralise – propagated by Hately et al. (1965), Fernandez-Rodriguez et al. (2009), Joshi et al. (2012), and Stojanovic (2013) – is refuted by accumulating CT evidence and should be replaced by the more accurate position that epiglottic mineralisation is uncommon, age- and sex-related, and mechanistically distinct from the endochondral ossification of hyaline laryngeal cartilages, its pathogenesis involving age-related elastic fibre loss and collagen replacement as documented by Serikawa et al. (2023). The index case demonstrates that almost complete plate-like mineralisation with 90° posterior angulation creates a measurable reduction in the hyoid-to-posterior-pharyngeal-wall distance (1.4–1.6 mm bilaterally) with potential relevance to swallowing mechanics, airway instrumentation, and – as hypothesised here for the first time – a fixed rigid-trapdoor mechanism for obstructive sleep apnea that may be anatomically and biomechanically distinct from the dynamic epiglottic collapse described in the DISE literature. These are morphological observations and mechanistic hypotheses: no clinical assessment of swallowing, airway, or sleep was available for this patient, and the proposed links require prospective clinical investigation. Future studies combining CT morphometry with polysomnographic data and standardised airway assessment in patients with documented epiglottic mineralisation are needed to test these hypotheses.

## Supplementary Information

Below is the link to the electronic supplementary material.

Supplementary Material 1: (ESM_1_sag_SUBMISSION.mp4): Sagittal cine computed-tomography-angiography reconstruction through the midline, demonstrating the almost completely mineralised epiglottis, its 90° posterior angulation, and its relationship to the hyoid body, pre-epiglottic space, and posterior pharyngeal wall.

Supplementary Material 2: (ESM_2_3D_SUBMISSION.mp4): Three-dimensional rotational reconstruction (360°) of the mineralised hyolaryngeal skeleton, confirming the epiglottic origin of the mineralised structure through its topographic relationship to the hyoid body and its spatial independence from the thyroid cartilage and the ossified stylohyoid chain. 1, mineralised epiglottis; 2, greater hyoid horn; 3, styloid process; 4, thyroid cartilage; 5, hyoid body.

## Data Availability

The DICOM datasets on which this case report is based are not publicly available owing to patient privacy constraints. All measurements and derived data supporting the findings of this study are contained within the article.
